# Lysosomes: multifunctional compartments ruled by a complex regulatory network

**DOI:** 10.1002/2211-5463.13387

**Published:** 2022-03-08

**Authors:** Jonathan Martinez‐Fabregas, Joaquin Tamargo‐Azpilicueta, Irene Diaz‐Moreno

**Affiliations:** ^1^ Instituto de Investigaciones Químicas (IIQ)—Centro de Investigaciones Científicas Isla de la Cartuja (cicCartuja) Universidad de Sevilla—CSIC Spain

**Keywords:** lysosomes, mTORC1, signalling pathways, STATs, TFEB, transcriptional regulation

## Abstract

More than 50 years have passed since Nobel laureate Cristian de Duve described for the first time the presence of tiny subcellular compartments filled with hydrolytic enzymes: the lysosome. For a long time, lysosomes were deemed simple waste bags exerting a plethora of hydrolytic activities involved in the recycling of biopolymers, and lysosomal genes were considered to just be simple housekeeping genes, transcribed in a constitutive fashion. However, lysosomes are emerging as multifunctional signalling hubs involved in multiple aspects of cell biology, both under homeostatic and pathological conditions. Lysosomes are involved in the regulation of cell metabolism through the mTOR/TFEB axis. They are also key players in the regulation and onset of the immune response. Furthermore, it is becoming clear that lysosomal hydrolases can regulate several biological processes outside of the lysosome. They are also implicated in a complex communication network among subcellular compartments that involves intimate organelle‐to‐organelle contacts. Furthermore, lysosomal dysfunction is nowadays accepted as the causative event behind several human pathologies: low frequency inherited diseases, cancer, or neurodegenerative, metabolic, inflammatory, and autoimmune diseases. Recent advances in our knowledge of the complex biology of lysosomes have established them as promising therapeutic targets for the treatment of different pathologies. Although recent discoveries have started to highlight that lysosomes are controlled by a complex web of regulatory networks, which in some cases seem to be cell‐ and stimuli‐dependent, to harness the full potential of lysosomes as therapeutic targets, we need a deeper understanding of the little‐known signalling pathways regulating this subcellular compartment and its functions.

## Graphical abstract

Lysosomes, considered for a long time as the cell’s recycling plant, are emerging as multifunctional signalling hubs involved in the regulation of multiple aspects of cell biology, from cell metabolism to immune responses. Furthermore, lysosomal dysfunction is nowadays accepted as the causative event behind several human pathologies. The essential role played by lysosomes rationalises the complex regulatory network controlling their activities.

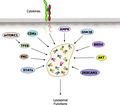

**Abbreviations**
AEP: asparagine endopeptidase
AMPK: 5′‐adenosine monophosphate (AMP)‐activated protein kinase
ATG13: autophagy‐related protein 13
ATG14: autophagy‐related protein 13
BAD: Bcl2‐associated agonist of cell death
BRD4: bromodomain‐containing protein 4
CaMKK2: calcium/calmodulin‐dependent protein kinase kinase 2
CCNB: cyclin B
CDK: cyclin‐dependent kinase
CLEAR: coordinated lysosomal expression and regulation
FoxP3: forkhead box protein 3
GSK3β: glycogen synthase kinase‐3 beta
IKK: IκB kinase
IL: interleukin
LKB1: serine/threonine protein kinase STK11
LM‐PCD: lysosomal mediated programmed cell death
MHC: major histocompatibility complex
MiTF: microphthalmia‐associated transcription factor
mTOR: mechanistic target of rapamycin
mTORC1: mechanistic target of rapamycin complex 1
MYC: Myc proto‐oncogene protein
NF‐κB: nuclear Factor kappa‐light‐chain enhancer of activated B cells
OSM: oncostatin M
PKB: protein kinase B (also known as AKT)
PKC: protein kinase C
pTEFb: positive transcription elongation factor b
RAPTOR: regulatory‐associated protein of TOR
STAT: signal transducer and activator of transcription
TFE3: transcription factor E3
TFEB: transcription factor EB
TFEC: transcription factor EC
TFIIH: transcription factor II H
TLR: toll‐like receptor
TNF: tumour necrosis factor
TSC1: tuberous sclerosis 1 protein
TSC2: tuberous sclerosis 2 protein
ULK1: Unc51‐like kinases 1
ZKSCAN3: zinc finger protein with KRAB and SCAN domains 3

## Lysosomes as multifunctional subcellular compartments

Through classical biochemistry approaches, Cristian de Duve was able to describe in the 1950's the presence of small subcellular compartments filled with hydrolytic enzymes, the lysosomes, and anticipate the importance of these tiny hydrolytic vacuoles [1]. However, for almost 60 years, lysosomes have been widely considered to be simple waste bags involved in the recycling of biopolymers (such as proteins, carbohydrates, nucleic acids, etc) into building blocks that can be reused by cells. Moreover, lysosomal genes have been regarded as housekeeping genes transcribed in a constitutive, unregulated fashion.

**Fig. 1:**
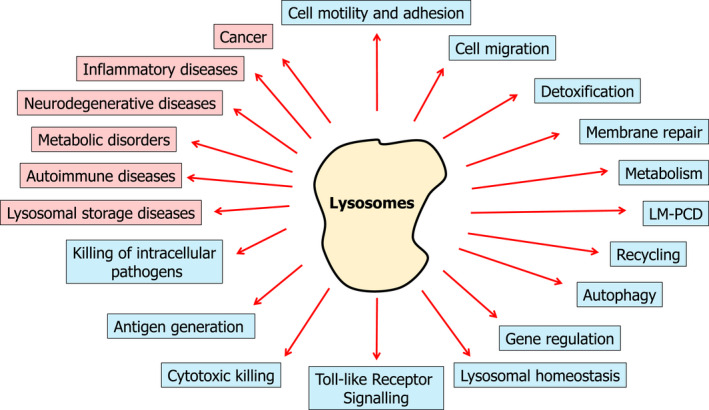
Lysosomes as multifunctional compartments. The main lysosomal functions described in the literature under physiological (in blue) and pathological (in red) conditions are presented.

Nowadays, lysosomes are highly recognized as multifunctional compartments involved in a plethora of biological processes under both physiological and pathophysiological conditions [2, 3] (Fig. 1). Under physiological conditions, lysosomes play key roles as regulators of multiple aspects of cell biology, including immune responses, membrane repair, cell adhesion and migration, regulation of transcriptional and translational events, and the integration of signals originating from different compartments to regulate cell metabolism [2]. The central role of lysosomes in the regulation of these biological processes rationalizes why lysosomal dysfunction represents a major causative event linked to the onset and progression of multiple human pathologies.

Under homeostatic conditions, lysosomes play a key role in the regulation of metabolism by controlling signalling pathways that switch the cell metabolism from anabolism to catabolism depending on the energetic status of the cell. Under these conditions (*e.g*., high levels of amino acids), lysosomes play a key role in the activation of mTOR signalling allowing the activation of anabolic (biosynthetic) pathways. On the contrary, during starvation, lysosomes play an essential role as the final recipients of the autophagic cargo, recycling unneeded material into individual building blocks that can be reused by the cell, thus allowing cell survival [4, 5].

Lysosomes also play key functions in the regulation of both the innate and adaptive immune responses. Lysosomal proteases (*e.g*., legumain/asparagine endopeptidase) are involved in the activation of toll‐like receptors (TLRs) (*i.e*., TLR7 and TLR9) that recognize microbial products, playing a critical role in innate and adaptive immunity [6, 7, 8]. Furthermore, pathogens that end up in the endo/lysosomal system (intracellular pathogens) can be killed by the hydrolytic enzymes located in lysosomes, providing the latter with defence functions during infection [9]. Moreover, lysosome‐related cytotoxic granules are critical for the elimination of virus‐infected host cells and cancer cells [10, 11]. Finally, lysosomes are also essential in the generation and presentation of antigens [12, 13]. Both extracellular and intracellular proteins can be delivered into the lysosomal compartment where they are degraded, leading to the generation of antigens that can then be loaded into major histocompatibility complex (MHC) molecules and presented to T cells to activate the immune response. The generation of antigens in the endo/lysosomal compartment is not only important for the presentation of exogenous antigens required for the immune response against pathogens but also in the display of self‐antigens in the development of self‐tolerance [12]. However, how the immune cells regulate their lysosomal activity to guarantee efficient antigen generation or to provide maximum activity to eliminate the infection is still poorly understood.

Furthermore, these tiny hydrolytic vacuoles have been shown to play critical roles in cell migration [14], adhesion and motility [2, 15, 16], detoxification [17], membrane repair [18], and other processes, all of which are carried out within the lysosomal compartment, isolated from the cytoplasm. However, extralysosomal functions of lysosomal hydrolases have recently started to emerge. In this regard, lysosomal hydrolases have been shown to play a key role in the regulation of Treg differentiation through the degradation of FoxP3 via legumain [19] or during chromosome segregation at cell division [20, 21]. One of these extralysosomal functions of the lysosomal hydrolases—their ability to promote cell death upon release from the lysosomal compartment—was already anticipated by Cristian de Duve. Several different groups have contributed to the elucidation of the role played by lysosomes in the onset and progression of this novel pathway of cell death, so‐called lysosomal‐mediated programmed cell death (LM‐PCD) [22, 23].

Additionally, lysosomes are also involved in different pathophysiological conditions, such as cancer [24, 25], rare inherited diseases [26] (*e.g*., lysosomal storage diseases), neurodegenerative diseases [27], inflammatory diseases [28], and autoimmune [29] and metabolic [30] disorders.

Altogether, the vast array of functions played by lysosomes under physiological conditions, ranging from cell homeostasis and metabolism to cell death and immune response, and their role in the onset and progression of several human pathologies, have made lysosomes emerge as a very interesting therapeutical target. However, how these tiny hydrolytic vacuoles are regulated in response to different stimuli remain only partly understood. To really exploit the full potential of lysosomes as therapeutic targets for the treatment of these different maladies, we need a deeper, more complete understanding of how lysosomes are regulated in response to different cellular stimuli and in different cell types, and how the different signalling pathways—those already described and the ones still waiting to be uncovered—integrate into a final cellular response.

## Coordinated regulation of lysosomal biogenesis and autophagy: the mTORC1/TFEB axis

**Fig. 2:**
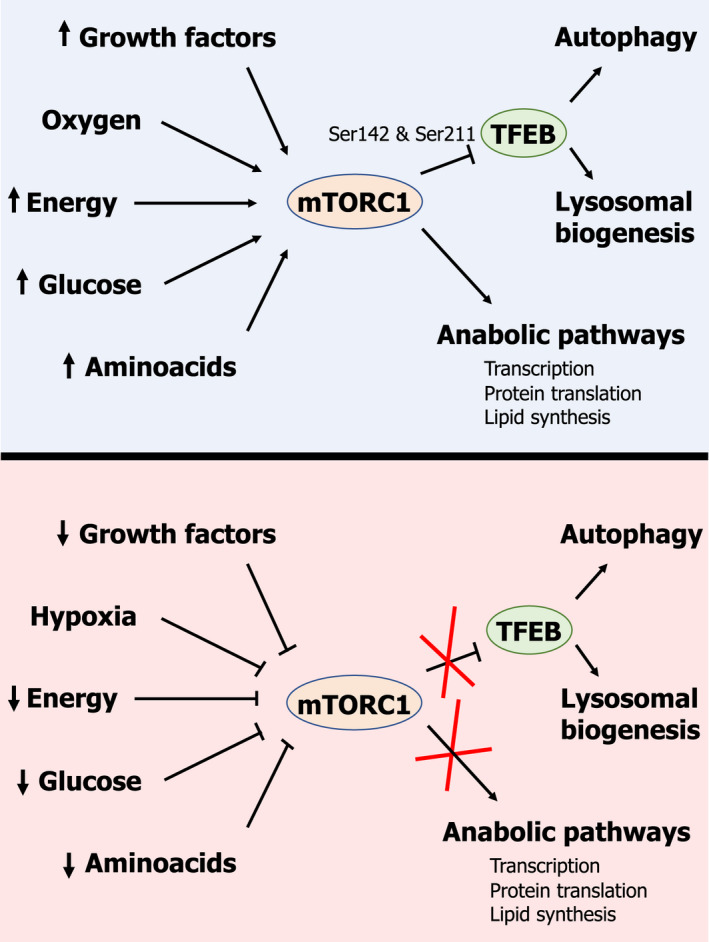
Schematic representation of the metabolic and physiological cues controlling the mTORC1/TFEB axis in their role as regulators of both autophagy and lysosomal biogenesis.

Seminal work performed by Ballabio's group began to elucidate how lysosomal genes are regulated in order to adjust their hydrolytic capacity in response to several intracellular and extracellular stimuli [31, 32] (Fig. 2). Although lysosomal genes were for a long time considered to be transcribed in a constitutive fashion, it is becoming clear that cells have the ability to check their lysosomal activity and energetic state and regulate the transcription of lysosomal genes in order to meet cellular needs. This pathway of lysosomal regulation relies on the mammalian target of rapamycin (mTOR) complex 1 (mTORC1) and the transcription factor EB (TFEB). mTOR is an evolutionarily well‐conserved Ser/Thr kinase, which plays a key role in the regulation of cell metabolism by integrating information from different sources—amino acid and glucose levels, hormones, growth factors, hypoxia or starvation—in order to regulate different aspects of cell physiology, such as cell growth, transcription, translation, stress response, gene expression, etc. [2] (Fig. 2). TFEB is a member of the microphthalmia‐associated family of the basic helix‐loop‐helix (b‐HLH) leucine zipper transcription factor (MiT/TFE) family of transcription factors, including MiTF, TFE3, TFEB, and TFEC [33]. Upon activation, TFEB translocates to the nucleus and binds to the coordinated lysosomal expression and regulation (CLEAR, also known as E‐boxes) elements (GTCACGTAC) enriched in the promoter region of numerous lysosomal and autophagic genes, activating the coordinated transcription of these two sets of genes [31, 32, 34]. Interestingly, other members of the MiT/TFE family have been shown to be able to regulate both lysosomal biogenesis and autophagy, demonstrating some overlap in their physiological function [35, 36, 37].

The activity of mTORC1 is highly regulated in response to various inputs, including energetic status, amino acid levels, and growth factors. Under favourable metabolic conditions (*e.g*., high amino acid levels, high ATP/ADP or ATP/AMP ratios, *etc*.) or upon stimulation with growth factors, mTORC1 is activated through different mechanisms that are extensively reviewed in the literature [4, 5, 38, 39, 40]. Once activated, mTORC1 performs its role as a regulator of the activity of multiple biosynthetic pathways, thus promoting cell growth and proliferation [4] (Fig. 2, upper panel). Furthermore, mTORC1 in its active state also inhibits autophagy and other catabolic pathways [4]. To this end, mTORC1 phosphorylates and inhibits key players in the regulation of autophagy, such as TFEB [32] and Unc51‐like kinases 1 (ULK1) [41, 42]. Active mTORC1 can phosphorylate TFEB on Ser142 and Ser211 located on the nuclear localization signal (NLS) of TFEB, leading to its cytosolic sequestration via 14‐3‐3 interaction [43]. On the contrary, under conditions of starvation (*e.g*., reduced levels of amino acids, low ATP/ADP or ATP/AMP ratios, *etc*.) or during growth factor withdrawal, mTORC1 becomes inactivated, leading to TFEB dephosphorylation [43]. Upon dephosphorylation, TFEB is released from 14‐3‐3 and then is translocated to the nuclei, where it activates the transcription of both lysosomal and autophagic genes by binding a specific DNA sequence (GTCACGTAC) present in the promoter region of both lysosomal and autophagic genes [31, 32, 43] (Fig. 2, lower panel). Concurrently, mTORC1 deactivation leads to the dephosphorylation and activation of ULK1, enabling it to phosphorylate and activate several autophagy‐related genes critical for autophagy initiation, such as Beclin1 and VPS34 [44]. This leads to coordinated shutdown of the main anabolic pathways and the activation of autophagy, in an attempt to allow cell survival.

## Energetic status: AMPK and regulation of lysosomes

**Fig. 3:**
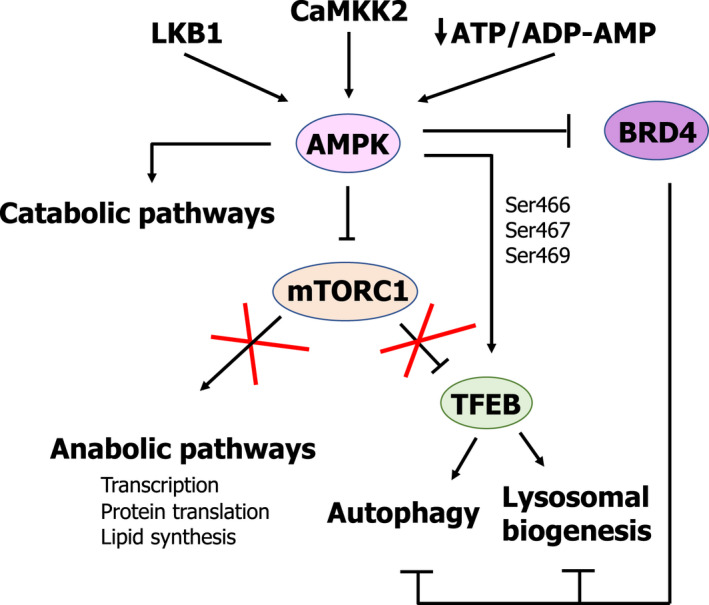
Role of AMPK as a positive regulator of lysosomal biogenesis and autophagy both indirectly, through mTORC1 inhibition, and directly, through phosphorylation and transcriptional activation of TFEB.

5′‐adenosine monophosphate (AMP)‐activated protein kinase (AMPK) is a key regulator of energy homeostasis [45] (Fig. 3). This protein is an evolutionarily well‐conserved Ser/Thr kinase comprising three different subunits: the α subunit (α1 and α2), which harbours the catalytic activity of the complex, the β subunit (β1 and β2), with a scaffolding role, and the γ subunit (γ1, γ2, and γ3), which has a regulatory function [45, 46]. AMPK acts as a regulator of energy homeostasis by integrating information from different sources, such as glucose deprivation and hypoxia, and regulating both anabolic and catabolic pathways [45, 46, 47]. AMPK activation under conditions of metabolic deprivation requires two sequential steps. First, the allosteric activation of AMPK by AMP. Under conditions of metabolic deprivation, the ratio ATP/ADP‐AMP is reduced; therefore, AMP can bind to AMPK, inducing a conformational change that allows for the phosphorylation and activation of its Ser/Thr kinase activity. Upon binding of AMP, the kinase domain of the α subunit of AMPK becomes exposed, making it susceptible to (a) phosphorylation on Thr172 and (b) activation by different upstream kinases in a second step (*e.g*., LKB1 and CaMKK2) [48, 49, 50, 51]. In this way, activation of AMPK under conditions of metabolic need leads to the inhibition of anabolic pathways and activation of degradative/catabolic pathways in an attempt to restore energy homeostasis.

Thus, AMPK and mTORC1 play opposite roles in the regulation of cell metabolism (Fig. 3). Under normal metabolic conditions, mTORC1 is active, while AMPK remains repressed, leading to the activation of biosynthetic/anabolic pathways (*e.g*., protein synthesis, gluconeogenesis, *etc*.) and inhibition of degradative/catabolic pathways (*e.g*., autophagy), thereby promoting cell growth and proliferation. On the other hand, under conditions leading to a low energetic status (reduced ratio ATP/ADP‐AMP), mTORC1 is inactivated, while AMPK becomes active, leading to the activation of catabolic pathways and the inhibition of anabolic ones, in an attempt to shut down unnecessary metabolic routes, thus promoting cell survival. Interestingly, AMPK has been shown to be able to inactivate mTORC1 through two different mechanisms: (a) by phosphorylating TSC‐2 at Ser1387 [52] and (b) by phosphorylating mTORC1 subunit RAPTOR at Ser792 [53]. Furthermore, both Ser/Thr kinases have been shown to phosphorylate TFEB, although they play opposite roles. Whereas mTORC1‐mediated TFEB phosphorylation on Ser211 leads to cytoplasmic retention through 14‐3‐3 protein interaction and inactivation [43], AMPK has also recently been shown to phosphorylate TFEB [54, 55]. In contrast to mTORC1, AMPK‐mediated Ser phosphorylation of TFEB on residues 466, 467, and 469 under conditions of metabolic starvation has been shown to play a critical role in the activation of its transcriptional activity [55] (Fig. 3). This dual regulation of TFEB by mTORC1 and AMPK is similar to the regulation of ULK1 [41], proving that the regulation of the activity of the lysosomal compartment is more complex and sophisticated than initially considered.

Furthermore, AMPK has also been reported to be involved in the regulation of autophagy and lysosomal biogenesis through the regulation of the bromodomain‐containing protein 4 (BRD4) (Fig. 3). BRD4, which is a member of the bromodomain and extraterminal (BET) family of transcription factors, has been shown to play a crucial role in the regulation of autophagy and the transcription of lysosomal genes [56]. BRD4 regulates cell growth and cell cycle progression by binding to acetylated histones and transcription factors, promoting the recruitment of complexes involved in the regulation of transcription, such as the mediator and the pTEFb complexes. More recently, BRD4 has been described to be involved in the regulation of a plethora of biological processes, ranging from the DNA damage response to memory formation [57].

Interestingly, BRD4 has been shown to play a key role in the inhibition of autophagy and the expression of lysosomal genes [56]. Under normal metabolic conditions, BRD4 represses the transcription of lysosomal and autophagy‐related genes by occupying their promoters. However, during starvation BRD4 dissociates from the promoter of autophagy and lysosomal genes through a mechanism involving AMPK and sirtuin‐1 (SIRT‐1) [56]. Moreover, the transcriptional activation of autophagy and lysosomal genes upon dissociation of BRD4 seem to be independent of TFEB, TFE3, and MiTF.

## PKC/GSK3β: a novel mTORC1‐independent ZKSCAN3‐mediated pathway of lysosomal regulation

**Fig. 4:**
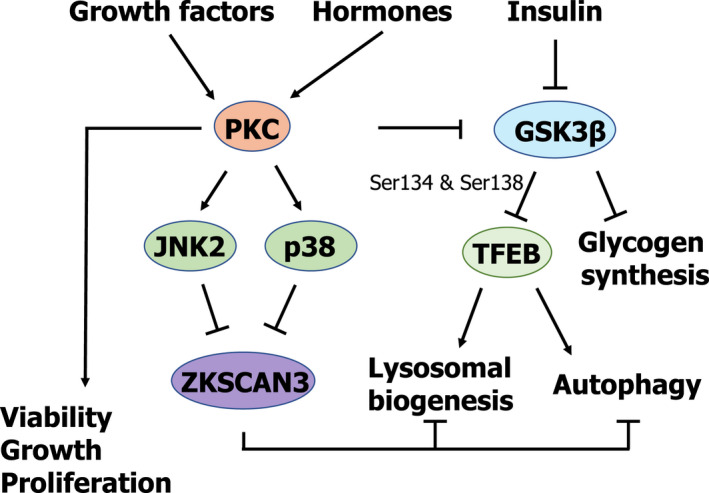
Schematic representation of PKC and GSK3β as mTORC1‐independent regulators of autophagy and lysosomal biogenesis.

Recent reports have identified ZKSCAN3 as a novel transcription factor involved in the regulation of lysosomal biogenesis and autophagy (Fig. 4). It has been shown that under normal metabolic conditions ZKSCAN3 localises in the nucleus, binding and repressing the transcription of lysosomal and autophagy genes by recognising a specific DNA sequence present in the promoter region of these genes ([GT][AG][AGT]GGGG) [58, 59]; this indicates that there is no direct competition between TFEB and ZKSCAN3 for binding to the promoter region of target genes. However, during starvation ZKSCAN3 relocates to the cytoplasm, while TFEB translocates to the nucleus and binds to the promoter region of lysosomal and autophagy genes, resulting in their transcription [58, 59].

As previously mentioned, the transcription factors TFEB and ZKSCAN3 play opposite roles in the regulation of lysosomal biogenesis and autophagy [31, 32, 58, 59], thus providing a double level of security in order to adequately regulate the anabolic and catabolic pathways to meet the cell’s metabolic needs (Fig. 4).

The molecular mechanisms involving protein kinase C (PKC) and glycogen synthase kinase‐3 β (GSK3β) in the coordinated regulation of both transcription factors have recently been elucidated [60] (Fig. 4). PKC refers to a family of Ser/Thr protein kinases comprised of several different isozymes extensively studied due to their key role in different human pathologies, namely cancer, diabetes, heart diseases, neurodegenerative diseases (*e.g*., Parkinson’s and Alzheimer’s) and autoimmune diseases [61, 62, 63, 64, 65]. PKCs are comprised of two well‐distinguished functional domains: the N‐term, which contains the regulatory region, and the C‐term, which contains the catalytic domain [66, 67]. PKCs are activated by growth factors, hormones, or neurotransmitters that, upon binding to their receptors, cause the activation of phospholipase C, which generates diacylglycerol, one of the most potent activators of PKCs [66, 67]. However, some PKC isoforms also require Ca^2+^ in addition to diacylglycerol to become active [66, 67].

On the other hand, GSK3β is a Ser/Thr kinase involved in the regulation of cell metabolism in response to insulin [68]. Activated GSK3β phosphorylates and inactivates glycogen synthase, promoting catabolic metabolism. However, upon binding of insulin to its surface receptor, GSK3β is phosphorylated and inactivated, allowing the activation of the glycogen synthase, and thus promoting anabolic metabolism.

As recently shown by Li et al. [60]., activation of PKC results in lysosomal biogenesis, concurrently leading to TFEB activation and ZKSCAN3 inactivation in an mTORC1‐independent fashion. On the one hand, activation of the PKCα and PKCδ isoforms results in the inactivation of GSK3β through phosphorylation at residues Ser9 and Ser21. In its phosphorylated state, GSK3β can no longer phosphorylate TFEB on residues Ser134 and Ser138. The phosphorylation of TFEB on Ser 134 and Ser138 has been shown to play a critical role in bringing TFEB to the lysosomal surface in close proximity to active mTORC1, thereby allowing mTORC1 to phosphorylate TFEB on Ser142 and Ser211, leading to TFEB sequestration in the cytosol by its interaction with 14‐3‐3 protein (Fig. 4). Thus, PKC activation leads to the activation of TFEB by blocking its lysosomal translocation and subsequent phosphorylation by mTORC1. On the other hand, PKC activation is also crucial in the regulation of ZKSCAN3 (Fig. 4). Once activated, PKC phosphorylates and activates JNK2 and p38, which are responsible for the phosphorylation of ZKSCAN3 on Thr153, leading to its cytoplasmic translocation [60]. Therefore, this PKC‐mediated, mTORC1‐independent pathway activates lysosomal biogenesis and autophagy by simultaneously activating TFEB, allowing its nuclear translocation and the transcriptional activation of lysosomal and autophagy genes, while at the same time releasing the block by inactivating and removing ZKSCAN3 from the nucleus (Fig. 4).

Interestingly, Ryu et al. [69] have recently shown that upon insulin withdrawal, GSK3β can also trigger autophagy initiation through direct phosphorylation of ULK1 on Ser405 and Ser415. How the cell is able to regulate this dual, opposing role of GSK3β (*i.e*., being able to block lysosomal biogenesis and autophagy through the phosphorylation of TFEB and to activate autophagy through the phosphorylation of ULK1 upon insulin withdrawal) remains poorly understood. This again highlights the enormous complexity of the regulation of the lysosomal compartment and autophagy required to guarantee the appropriate metabolic response to extracellular and intracellular inputs.

## AKT: another modulator of TFEB

**Fig. 5:**
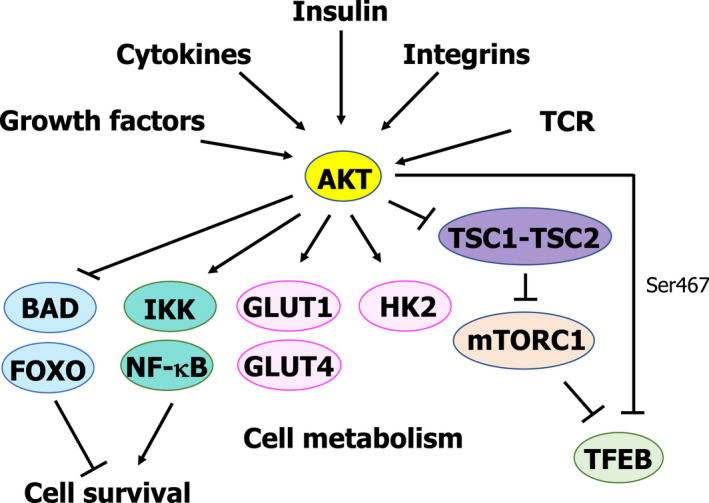
Role of AKT as a negative regulator of lysosomal biogenesis and autophagy independently of mTORC1 activity.

Protein kinase B (PKB), also known as AKT, is a family of Ser/Thr kinases comprised of three different isoforms named AKT1, AKT2, and AKT3 [70]. AKT integrates signals originating at different levels (*e.g*., growth factors or metabolic cues), regulating several physiological processes such as proliferation, glucose metabolism, transcription, apoptosis, and cell growth through direct phosphorylation of key regulators of these signalling pathways [70] (Fig. 5).

As previously mentioned, AKT has been shown to regulate apoptosis and promote cell survival by not only phosphorylating the proapoptotic protein BAD on Ser136, thus inhibiting its proapoptotic function [71], but also through the phosphorylation and inactivation of the proapoptotic FOXO proteins [72]. Furthermore, AKT promotes cell survival by activating the axis IKK (IκB kinase)/nuclear factor kappa‐light‐chain enhancer of activated B cells (NF‐κB), thus promoting the transcription of prosurvival genes [73, 74] (Fig. 5).

AKT is also known to regulate cell metabolism at different levels. AKT regulates glucose metabolism by directly increasing glucose uptake through the activation of glucose transporters such as GLUT1 and GLUT4 [75, 76, 77] and also by stimulating glycolysis through direct phosphorylation and activation of hexokinase 2 [78] (Fig. 5). Furthermore, AKT has been shown to control cell proliferation and metabolism through the regulation of mTORC1 activity [79]. In response to growth factors or cytokines, AKT has been shown to be able to phosphorylate the TSC2 protein, leading to the inactivation of the TSC1‐TSC2 complex, which plays an inhibitory role on mTORC1 [79] (Fig. 5). Thus, upon activation induced by growth factors, AKT can activate mTORC1, therefore promoting cell growth and proliferation, and inhibiting TFEB.

Quite recently, another layer of complexity has been uncovered in the role played by AKT in the regulation of metabolism. AKT can regulate TFEB activity independently of mTORC1 by directly phosphorylating TFEB at Ser467, thus blocking its nuclear translocation, and in this way, controlling lysosomal biogenesis and autophagy, as shown by Palmieri et al. [80] (Fig. 5). Furthermore, they were able to show in a mouse model of Batten disease—a neurodegenerative disease characterised by intralysosomal storage—how the inhibition of AKT with the well‐established inducer of autophagy trehalose or with specific AKT inhibitors leads to activation and nuclear translocation of TFEB. This treatment led to an increase in mouse survival and reduced neuropathology, as well as to reduced intralysosomal accumulation of proteolipid aggregates.

As previously mentioned, TFEB phosphorylation Ser467 by AKT has been described to block TFEB nuclear translocation independently of mTORC1 in a Batten disease mouse model [80] (Fig. 5). However, as recently shown [55] under conditions leading to mTORC1 inactivation (e.g., starvation, pharmacological inhibition of mTORC1, etc.), TFEB cellular location depends on mTORC1‐mediated phosphorylation of TFEB on Ser142 and Ser211, but its fully transcriptional activity requires AMPK‐mediated phosphorylation of TFEB on Ser466, Ser467, and Ser469 (Fig. 3). How the phosphorylation of the same Ser residue (*i.e*., Ser467) on TFEB can have different outcomes remains unanswered. However, this apparently contradictory result could be explained by differences in the models used. Palmieri et al. [80] demonstrated that AKT can regulate TFEB activity by blocking its nuclear translocation in a Batten disease mouse model and that AKT inhibition attenuated neuropathology observed in the model. On the other hand, Paquette et al. [55] showed that stimuli leading to mTORC1 inhibition and AMPK activation (starvation, mTORC1 inhibition, etc.) triggered nuclear translocation of TFEB to the nucleus (mediated by Ser142 and Ser211 dephosphorylation). However, the full transcriptional activity of TFEB requires TFEB phosphorylation on Ser466, Ser467, and Ser469. Therefore, further investigation is required to fully address the functional difference observed upon Ser467 phosphorylation of TFEB.

## CDKs—controlling lysosomal biogenesis and autophagy throughout the cell cycle

Cyclin‐dependent kinases (CDKs) have recently started to emerge as mTORC1‐independent regulators of both lysosomal biogenesis and autophagy throughout different stages of the cell cycle.

Cyclin‐dependent kinases are a family of Ser/Thr kinases whose activity requires the binding of specific cyclins. Although initially identified as regulators of the cell cycle by controlling the progression of the cell through the different stages of the cell cycle [81], CDKs are now known to regulate multiple aspects of cell biology, such as transcription (*e.g*., CDK7, CDK8, and CDK9 as part of the transcription factor II H (TFIIH) [82], mediator [83, 84] and the positive transcription elongation factor b (pTEFb) [85], respectively) and mRNA processing [86].

**Fig. 6:**
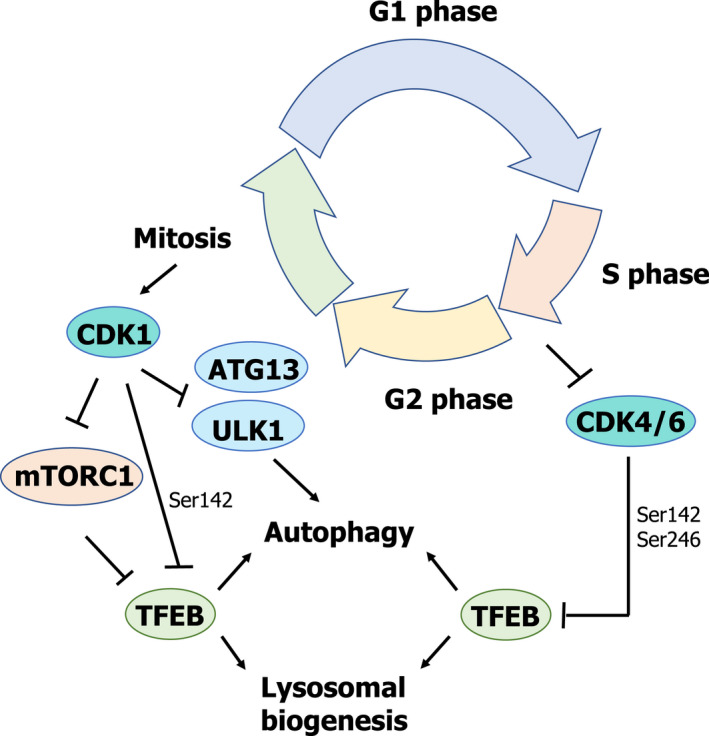
Scheme summarizing the role of CDKs as novel regulators of lysosomal biogenesis and autophagy, independently of mTORC1 activity.

Besides these well‐established functions, recent reports elucidate a novel role for different members of the CDK family as regulators of lysosomal biogenesis and autophagy (Fig. 6). As recently shown by Odle et al. [87], the regulation of both autophagy and lysosomal biogenesis seems to differ at different stages during the cell cycle. As previously described, mTORC1 is responsible for the phosphorylation of different key players involved in the activation of both autophagy and lysosomal biogenesis (*i.e*., ATG13, ATG14, ULK1, and TFEB) during interphase and under normal metabolic conditions. However, the picture seems to be completely different depending on the stage of the cell cycle, specifically during mitosis. The cell cycle is usually divided into four different phases: G1 (cell growth), S (DNA synthesis), G2 (growth and preparation for cell division), and M (mitosis or cell division). During mitosis, the complex cyclin B (CCNB)–CDK1 takes over from mTORC1 in the regulation of both lysosomal biogenesis and autophagy [87]. During this stage, autophagy is indeed repressed, but through an mTORC1‐independent pathway. During mitosis, CDK1 phosphorylates RAPTOR, leading to the dissociation of mTORC1 from the lysosomes by blocking the interaction of mTORC1 with RAG proteins, thus rendering mTORC1 inactive [88] (Fig. 6). Under these conditions, autophagy should be active, but the complex CCNB‐CDK1 takes over mTORC1 function by phosphorylating ATG13 (Ser259), TFEB (Ser142) and ULK1 (Ser758) on the same positions regulated by mTORC1, thereby activating their well‐described roles as repressors of autophagy and lysosomal biogenesis [87] (Fig. 6).

Furthermore, as recently reported by Yin et al. [89], CDK4/6 can also regulate lysosomal biogenesis and autophagy at different stages of the cell cycle. They were able to show that CDK4/CDK6 interacts with TFEB and TFE3 in the nucleus, leading to their phosphorylation (TFEB at Ser142 and TFE3 at Ser246) and subsequent inactivation by inducing their nucleus‐to‐cytoplasm translocation (Fig. 6). However, during the S and G2/M phases, there is an increase in the lysosomal content of cells. The authors were able to unveil a novel link between CDK4/6 activity and the regulation of lysosomal biogenesis and autophagy during these phases of the cell cycle. During the S and G2/M phases, there is a decrease in the levels of cyclin D, which is required for CDK4/6 activity, therefore blocking TFEB and TFE3 phosphorylation by CDK4/6 and promoting lysosomal biogenesis (Fig. 6).

## STAT family: regulating lysosomes from cell death to homeostasis

**Fig. 7:**
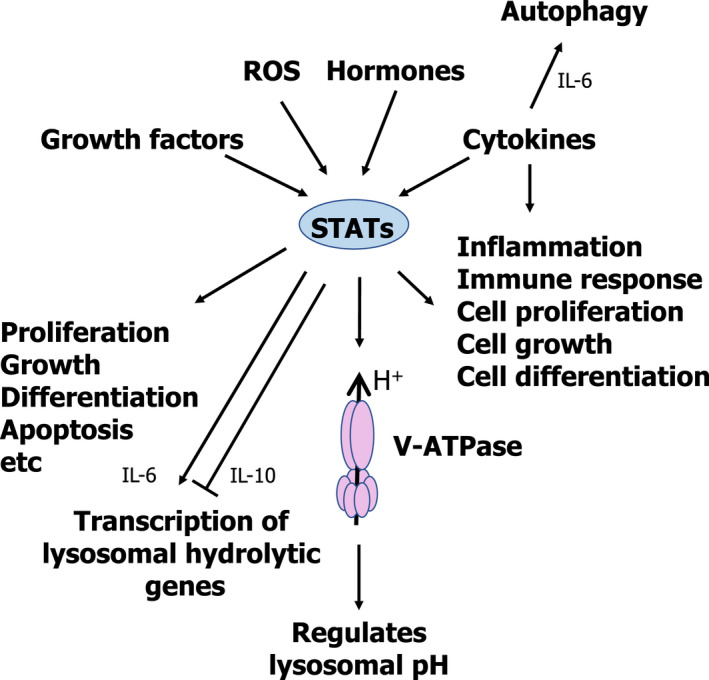
Schematic representation of the role played by cytokines and STATs as regulators of the lysosomal compartment.

Recent reports have started to elucidate the role played by different members of the signal transducer and activator of transcription (STAT) family of transcription factors in the regulation of the lysosomal compartment [22, 90, 91, 92] (Fig. 7). Seven different members of the STAT family have been identified in humans: STAT1, STAT2, STAT3, STAT4, STAT5A, STAT5B, and STAT6 [93, 94]. Initially identified in the early 1990s for their role in cytokine signalling and interferon‐mediated antiviral response, they are nowadays regarded as multifunctional proteins involved in the regulation of multiple aspects of cell biology (Fig. 7). Upon binding of different molecules (cytokines, hormones, growth factors) to their extracellular receptor, STATs participate in signal transduction into the nucleus by regulating the transcriptional response. STATs have been shown to be essential players in the regulation of cell proliferation and cell growth, differentiation, apoptosis, and regulation of the immune response, among other processes [93, 94].

The role played by different members of the STAT family in the regulation of the lysosomal compartment has started to be elucidated quite recently. In 2013, Brignull et al. [95] were able to show that lysosomal genes can be clustered into three independent groups, suggesting that the genes in each group can be regulated co‐ordinately, but independently from the other groups [95]. Cluster 1 mainly included genes related to the lysosomal membrane, while Cluster 2 mainly included genes encoding the different subunits of the vacuolar H^+^ ATPase and components of the endo/lysosomal trafficking machinery. Finally, Cluster 3 included genes encoding the lysosomal hydrolases and genes essential for antigen presentation and transport. Thus, it seems that cells have evolved a complex lysosomal regulatory network, allowing for the independent regulation of different aspects of the lysosomal compartment.

Brignull et al. [95] also identified those transcription factors which could potentially be responsible for the regulation of these different sets of genes. Interestingly, different members of the STAT family were identified as potential regulators of these genes; in particular, STAT2, STAT3, and STAT6 were proposed as potential regulators of cluster 3 of lysosomal genes. Furthermore, the same authors were able to experimentally show that STAT6 [activated via interleukin‐4 (IL‐4) stimulation] is indeed able to regulate lysosomal hydrolases in mouse macrophages.

The capacity of STAT3 to regulate lysosomal hydrolases had been recently reported [22, 90, 91, 92]. The key role played by STAT3 during the onset and progression of lysosomal‐mediated programmed cell death was recently reported. During postlactational mammary gland involution, oncostatin M (OSM) and STAT3 have been shown to play a key role in the upregulation of lysosomal proteases (*e.g*., Cathepsins B and L) and also in the control of the lysosome‐mediated, mitochondria‐independent pathway of cell death [22, 90, 91], different to the classical apoptotic pathway of cell death [96, 97, 98, 99]. Furthermore, STAT3 has recently been reported as a regulator of lysosomal homeostasis [92]. Under conditions of lysosomal stress, with excessive accumulation of lysosomal substrates or reduced hydrolytic capacity of the lysosomes (*e.g*., inhibition of proteolytic activities), STAT3 is phosphorylated and activates the coordinated transcription of lysosomal content (hydrolases, such as proteases and glycosidases), without modifying the number of lysosomes. This response is aimed at increasing the lysosomal hydrolytic capacity to restore the homeostatic balance [92] (Fig. 7).

Beyond its role as a transcription factor involved in the regulation of the expression of lysosomal genes, STAT3 has been shown to regulate intracellular pH. Liu et al. [100] showed that under conditions in which the intraluminal pH of the lysosomes is neutralized, leading to the acidification of the cytosolic pH (for example, by inhibiting the V‐ATPase (Bafilomycin A or Concanamycin) [101, 102, 103, 104] or by directly sequestering H^+^ in the lysosomal compartment (*e.g*. chloroquine) [105]; or through the acidification of the cytosolic pH, for example, by using inhibitors of the Na^+^/H^+^ exchanger (*i.e*., EIPA or cariporide) [106]), STAT3 shows a punctate pattern by specifically interacting with the lysosomal V‐ATPase complex. Liu et al. [100] were able to show that the interaction of STAT3 with the V‐ATPase requires the coiled‐coil domain of STAT3. Furthermore, they were able to show that interaction of STAT3 with the lysosomal V‐ATPase leads to an increase of the ATPase activity of the V1 domain within the V‐ATPase complex, thus acidifying the lysosomal pH and neutralizing the cytosolic pH, thus allowing for the quick recovery of the homeostatic balance and permitting the proper functioning of the lysosomal compartment (Fig. 7).

## Cytokines

Cytokines are small polypeptides secreted by a variety of different cell types that play key roles in the regulation of the immune system by acting in an autocrine (*i.e*., acting on the cell releasing the cytokine) or a paracrine (*i.e*., acting on a different cell) fashion [107, 108, 109]. One of the main features of cytokines is pleiotropy, as they have been shown to play multiple roles from controlling cell growth and proliferation to cell differentiation and survival. Cytokines are organized in three different families based on their mechanism of signalling and on structural aspects of both the cytokines and their receptors [108, 109].

Type I cytokines show a four α‐helical bundle structure, and their receptors are devoid of an intrinsic tyrosine kinase domain; therefore, they rely on the Janus family of kinases (JAK1, JAK2, JAK3, and TYK2) to phosphorylate downstream targets. Upon binding to their cognate receptors, these cytokines signal through different members of the STAT family of transcription factors. This group includes interleukin 2 (IL‐2), IL‐4, interleukin 6 (IL‐6), OSM, and the leukaemia inhibitory factor (LIF), among others.

Type II cytokines comprise type I and type II interferons and the interleukin 10 (IL‐10) family of cytokines. Similar to type I cytokines, type II cytokines also depend on the JAK family of kinases and the STAT family of transcription factors to transduce their signalling.

Finally, a third group of cytokines, structurally quite distinct from type I and type II cytokines, includes the tumour necrosis factor (TNF), interleukin 1 (IL‐1), and interleukin 17 (IL‐17) families of cytokines. All these cytokines use receptors containing a tyrosine kinase domain in their intracellular region, and thus, they are independent of the JAK family of kinases.

For a long time, cytokines have been shown to regulate lysosomal activity at different levels. As previously mentioned, IL‐4 can regulate the expression of genes coding for lysosomal hydrolases, thus controlling lysosomal activity without the synthesis of new lysosomes through the activation of STAT6 [95]. OSM has also been shown to directly regulate the transcription of lysosomal proteases in a STAT3‐mediated manner during the activation of LM‐PCD throughout the involution of the mammary gland [22, 90, 91]. Other cytokines have also been linked to the regulation of lysosomal activity. In the early 1990s, there were several reports that IL‐6 plays an essential role during muscle atrophy by controlling lysosomal activity through the transcriptional regulation of both cathepsin B and L in a mouse model [110, 111, 112]. More recently, Hop et al. [113] were able to show that IL‐6 promotes the clearance of *Brucella abortus* in mouse macrophages by regulating the bactericidal activity of their lysosomes. Specifically, they were able to show that IL‐6 can promote bacterial killing through regulation of the transcription of several lysosomal genes, including lysosomal proteases and hydrolases, such as cathepsin D, cathepsin H, cathepsin Z, hexosaminidase A, and hexosaminidase B, among others. Furthermore, IL‐6 has been shown to promote autophagy to protect pancreatic β cells from apoptosis [114]. As shown by Linnemann et al. [114], IL‐6 stimulation leads to the activation of AMPK, inhibition of mTORC1, and activation of AKT, thereby promoting autophagy. In contrast to IL‐6, a well‐described proinflammatory cytokine able to induce the transcription of lysosomal genes [113], IL‐10, an antiinflammatory cytokine, has been recently shown to inhibit the expression of several lysosomal genes [115]. As described by Hop et al., IL‐10 blocks lysosome‐mediated clearance of *Brucella abortus* in mouse macrophages by negatively regulating the transcription of several lysosomal hydrolases, such as hexosaminidase B, alpha‐galactosidase A, cathepsin A, cathepsin D, and cathepsin L, among others (Fig. 7).

## MYC

In addition to the transcription factors previously described as regulators of the lysosomal compartment, another player has recently been added to the picture. c‐MYC is another member of the b‐HLH leucine zipper class of transcription factors, which also regulates transcription by binding to the E‐boxes located in the promoter region of the target genes [116]. c‐MYC plays a key role as a regulator of cell metabolism [117], the cell cycle [118], and cell growth and proliferation [119]. As recently reported [120], c‐MYC is able to occupy the promoter region of autophagy and lysosomal genes by binding to the same E‐boxes or CLEAR motifs used by the MiT/TFE family of transcription factors, thereby inhibiting the expression of autophagy and lysosomal genes. The authors propose that this novel mechanism could have important implications during cell differentiation: in pluripotent stem cells, c‐MYC would remain bound to the promoter region of these genes, maintaining cells in their undifferentiated state, while during cell differentiation, the levels of c‐MYC are reduced, thus releasing the promoter regions and allowing the binding of the MiT/TFE transcription factors.

## Conclusions

Christian De Duve was able to realize the potential role that lysosomes, small subcellular compartments filled with a host of hydrolytic enzymes and properly isolated from the cellular cytosol through the lysosomal membrane, could play in the regulation of biological processes. However, for more than 50 years, lysosomes have been widely considered to be mere recycling compartments, involved in the degradation of old, damaged biopolymers, thus providing building blocks (amino acids, nucleotides, etc.) that can be reused by the cell to synthesise new molecules. Only recently, lysosomes have started to emerge as key signalling hubs involved in the regulation of several aspects of cell biology, thus explaining why lysosomal dysfunction plays a critical role in several pathophysiological conditions such as cancer, neurodegenerative diseases, and lysosomal storage diseases. Nowadays, lysosomes are accepted to be master regulators of cell metabolism, through the regulation of the mTORC1 complex and the activation of autophagy during starvation.

Lysosomes also play a key role in the generation of immune information. Extracellular and intracellular proteins are all delivered to the lysosomes. In the lysosomal lumen, these proteins are digested by lysosomal proteases, generating antigens to be presented through the MHC complex to T cells. Thus, lysosomes play a key role not only in the activation of the immune response against pathogens, but also in the generation of self‐tolerance by displaying self‐antigens. Moreover, lysosomal proteases have been shown to regulate both the innate and adaptive immune response through the activation of toll‐like receptors (TLRs). Finally, lysosomes are also essential for the killing of intracellular pathogens and the elimination of virus‐infected cells and cancer cells.

Beyond these functions, lysosomes are involved in the regulation of adhesion, motility and migration, membrane repair, detoxification, etc. All these functions happen in the lysosomal lumen, isolated from the cytosol. Interestingly, extralysosomal functions of the lysosomal proteases have begun to be reported: activation and progression of LM‐PCD, AEP‐mediated neurotoxicity through the cleavage and aggregation of Tau, regulation of mitosis, regulation of T‐cell differentiation through the regulation of FoxP3 levels by AEP activity, and others.

Taking all this into account, it does not come as a surprise that lysosomes play critical roles in the onset and progression of different pathophysiological conditions, such as cancer, neurodegenerative diseases, rare inherited diseases (*e.g*., lysosomal storage diseases), metabolic disorders, and inflammatory diseases.

The plethora of functions regulated by the lysosomal compartment justifies the complex network of intracellular and extracellular signals, kinases, and transcription factors (both activators and repressors) that has started to emerge. However, our current knowledge of lysosomal biology is still limited, and many questions remain unanswered. Lysosomes have been shown to be involved in organelle‐to‐organelle contacts with the mitochondria, the endoplasmic reticulum, and the nucleus. However, the role played by such subcellular compartment interactions remains to be elucidated, and their possible role in the regulation of lysosome‐controlled metabolism unexplored. The functions of some lysosomal hydrolases outside of the lysosomal compartment have begun to be elucidated; yet, some questions remain open: (i) how are these lysosomal proteases secreted in a controlled fashion? (ii) which biological processes are controlled by these hydrolases upon release? Furthermore, a vast majority of the proteins associated with the lysosomal membrane and lysosomal trans‐membrane proteins have unknown functions. Whether these proteins contribute to the nutrient‐sensing capacity of the lysosomes, which nutrients are detected by these proteins, and which signalling pathways engaged by them—one of those already described or new ones waiting to be identified—all remain to be determined. The role played by cytokines in the regulation of the immune response through control of lysosomal activity, and how different cytokines can regulate lysosomes in opposite ways though using the same signalling molecules, are also questions waiting to be answered. Are cytotoxic T cell granules and other lysosome‐related organelles regulated in a similar way as lysosomes, or do they have specific signalling pathways, allowing the cell to regulate them independently? Furthermore, the hierarchy of the different signalling pathways controlling lysosomes and how they are all properly integrated into the signalling machinery of the cell in order to activate the proper metabolic response remains poorly understood. How does the cell control the existence of different lysosomal compartments that exhibit differences in hydrolase content, pH, positioning, shape, etc? Are they controlled through different signalling pathways? Do they play unique roles?

Our current knowledge of lysosomal biology allows us to work on the treatment of different lysosomal‐related pathologies. For years, enzyme replacement (supplying the defective lysosomal enzyme) or substrate reduction (reducing the accumulation of undigested substrates due to the lack of a given lysosomal enzyme) have been used for the treatment and amelioration of the symptoms associated with LSDs. The use of elements able to destabilize the lysosomal membrane, thus leading to the leakage of lysosomal hydrolases into the cytosolic compartment, are being tested as inducers of cell death in cancer cells as potential therapeutic agents. Expanding our basic knowledge of lysosomal biology and obtaining a more detailed picture of how the lysosomal compartment is regulated in different cell types and in response to different stimuli or under different physiological conditions will allow us to design better therapies for the treatments of several pathologies linked to lysosomes.

## Conflict of interest

The authors declare no conflict of interest.

## Author contributions

JMF conceived and wrote the manuscript. IDM and JTA drafted and edited the manuscript. IDM managed projects and acquired funding.
